# Past, present and future of spike sorting techniques

**DOI:** 10.1016/j.brainresbull.2015.04.007

**Published:** 2015-10

**Authors:** Hernan Gonzalo Rey, Carlos Pedreira, Rodrigo Quian Quiroga

**Affiliations:** a Centre for Systems Neuroscience, University of Leicester, 9 Salisbury Road, Leicester LE1 7QR, UK; b Department of Experimental Psychology, University of Oxford, Tinbergen Building, 9 South Parks Road, Oxford OX1 3UD, UK

**Keywords:** Spike sorting, Extracellular recordings, Modeling, On-chip applications, Multielectrode recordings

## Abstract

Spike sorting is a crucial step to extract information from extracellular recordings. With new recording opportunities provided by the development of new electrodes that allow monitoring hundreds of neurons simultaneously, the scenario for the new generation of algorithms is both exciting and challenging. However, this will require a new approach to the problem and the development of a common reference framework to quickly assess the performance of new algorithms. In this work, we review the basic concepts of spike sorting, including the requirements for different applications, together with the problems faced by presently available algorithms. We conclude by proposing a roadmap stressing the crucial points to be addressed to support the neuroscientific research of the near future.

## 1 Introduction

**Fig. 1:**
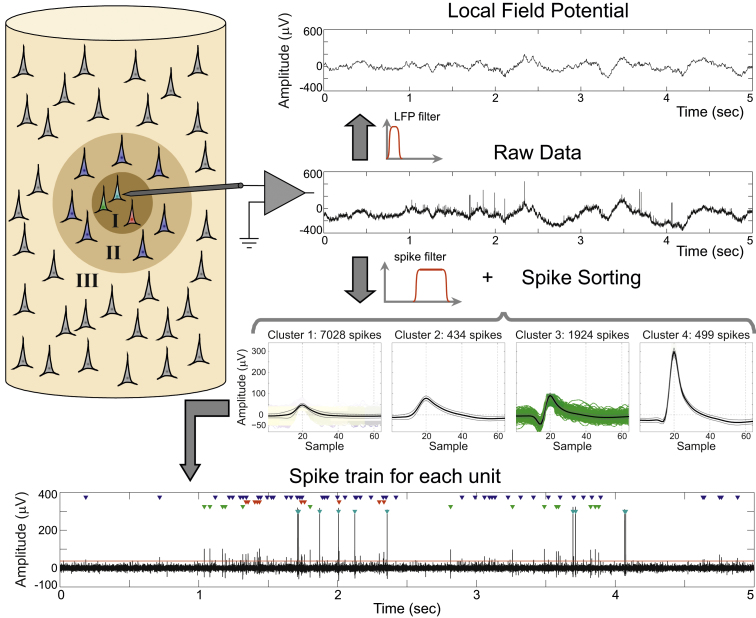
From extracellular recordings to spike trains. Example of an extracellular recording (raw data) from the human right entorhinal cortex. The low frequency content of that signal is associated with the local field potential (between 1 and 100 Hz in this example). Within the higher frequency content (between 300 and 3000 Hz in this example; black trace in bottom panel) there is a superposition of several effects. Neurons in zone III (more than ∼140 μm away from the tip of the electrode) contribute to the background noise, so their spikes cannot be detected. The neurons in zone II generate spikes larger than the background noise, but they cannot be separated into different units, thus being associated to the multiunit activity (cluster 1). Finally, the neurons in zone I (less than ∼50 μm away from the tip of the electrode) have even larger spikes, and sorting algorithms allow us to assign the recorded spikes to the different neurons that generated them (clusters 2–4), hence the so-called single unit activity. The sequence of spikes associated to each cluster is called a spike train. In the bottom panel, the time of occurrence of each spike is marked with a triangle color-coded according to the isolated clusters.

Monitoring the activity of single neurons in vivo is the basis for understanding the brain mechanisms supporting behavior. Following this principle, electrophysiologists have been recording the activity of neurons for decades. The cornerstone of these recordings is the placement of electrodes in brain tissue to register the extracellular activity of neurons. In these recordings the electrode is placed in the space between neurons, as opposed to other techniques such as *patch clamp* or *intracellular* recordings, in which the electrode is attached to one cell. The electrical potential changes measured at the electrode tip reflect current flows in the extracellular medium. The recording and data processing steps are described in Fig. 1. Basically, the recorded data is lowpass filtered to obtain the so called Local Field Potentials (LFPs), which reflect the dynamics of the neural tissue surrounding the electrode (around 1 mm in diameter). LFPs are generated by the input currents from the dendrites of the surrounding neurons and they are prominent in tissues where the cell bodies are (partially) aligned creating coherent dipoles in the recorded medium (Buzsáki et al., 2012). By bandpass filtering the signal, we obtain the activity of a few neurons close enough to the electrode plus background activity elicited by neurons further away from the tip (black trace in the bottom panel of Fig. 1).

In the recorded bandpass filtered signal, the activity of different neurons is superimposed and it is important to extract the identities of the spikes corresponding to different neurons. In principle, the spikes fired by a neuron recorded in a given electrode have a particular shape. This is mainly determined by the morphology of its dendritic tree, the distance and orientation relative to the recording site, the distribution of ionic channels and the properties of the extracellular medium (Gold et al., 2006). The detected spikes are grouped into different clusters based on their shapes in a process known as *Spike Sorting* (Quian Quiroga, 2007). Each cluster is then associated to a single unit (neuron), but some shapes cannot be separated due to a low signal to noise ratio, leading to a cluster associated with multiunit activity (Fig. 1). The multiunit cluster is formed by the superposition of different spikes, it has a relatively low amplitude and violates the refractory period of single neurons—i.e., spikes appear within less than 2.5 ms (Quian Quiroga, 2012a). By combining extracellular recordings and spike sorting methods we can isolate the activity of a few units per electrode for a period of time that ranges from a few hours, in the case of acute recordings in which the electrodes are lowered into cortex in each recording session, to months or even years in the case of stable chronically implanted electrodes (Homer et al., 2013).

The importance of spike sorting is stressed by the fact that close-by neurons—whose firing is picked up by the same electrode—can fire in response to different things and therefore, it might be crucial to know which spike corresponds to which neuron. This is the case, for example, in the human or rat hippocampus, where nearby neurons fire to unrelated concepts in the first case (Rey et al., 2014) and to distant place fields in the latter (Redish et al., 2001). But even when nearby neurons have similar responses (e.g., in primary visual cortex), it is important to distinguish them and observe their individual tuning properties, firing characteristics, relationship with other neurons and local field potentials, etc. In these cases, single neuron activity can reflect well-established processes like lateral inhibition and excitatory-inhibitory competition between nearby units. Accurate labeling of individual neurons is also crucial to study connectivity patterns of close-by neurons, or to study the topographical organization of a given area and discriminate the responses of nearby units (Quian Quiroga, 2007). In addition, spike sorting is essential for the identification of sparsely firing neurons that have, for example, been related to memory processes (Quian Quiroga, 2012b, Rey et al., 2014). The critical characteristic of these units is their very low baseline activity, thus been easily missed without proper sorting because of the more dominant firing of other nearby neurons with larger firing rates (Rey et al., 2014).

For all these reasons, the development of spike sorting algorithms has always been a key issue in the analysis of electrophysiological recordings. Ever since the beginning of extracellular recordings, several efforts have been made to develop such techniques (Abeles and Goldstein, 1977, Lewicki, 1998). In general, the number of simultaneously recorded neurons has grown exponentially since the 1950s, doubling every 7 years, and currently allowing electrical observation of hundreds of neurons at sub-millisecond timescales (Stevenson and Kording, 2011). However, this improvement should be largely attributed to the development of better recording techniques—i.e., the use of tetrodes, namely, 4 recording sites typically 25–50 μm apart (Gray et al., 1995), followed by polytrodes, namely, one or more columns with 8–64 channels spaced 50–70 μm (Blanche et al., 2005, Buzsáki, 2004, Csicsvari et al., 2003)—and has so far not being matched by analogous improvements in spike sorting algorithms. The lagging in the development of optimal spike sorting algorithms (see Section 5) is becoming critical, considering that within the next 10 years we will likely witness major changes in the electrode design and the capabilities of data acquisition systems, taking the number of recording sites to thousands (Alivisatos et al., 2013). Unfortunately, current spike sorting algorithms are a long way off to face the challenge of processing efficiently and reliably such a high volume of data. The amount and complexity of the data (number of channels times number of samples per second plus the information derived from the spatial configuration of the recording sites) to be produced by the next generation of probes is too large to be handled by researchers in a supervised way. Hence, the only viable option to deepen our understanding of the brain through the technology already available to us is the development of easy-to-use and properly validated tools for fully automatic spike sorting (Einevoll et al., 2012).

In this review we provide a general overview of the principles of spike sorting, starting by framing the problem and presenting classic solutions spread across the scientific community. Then, we will review constrains and limitations faced by these solutions and we will propose a unified framework for testing and comparing the performance of different algorithms. Finally, we outline the lines to be followed in the next few years to provide adequate support to the new generation of recording probes.

## 2 Current spike sorting strategies

**Fig. 2:**
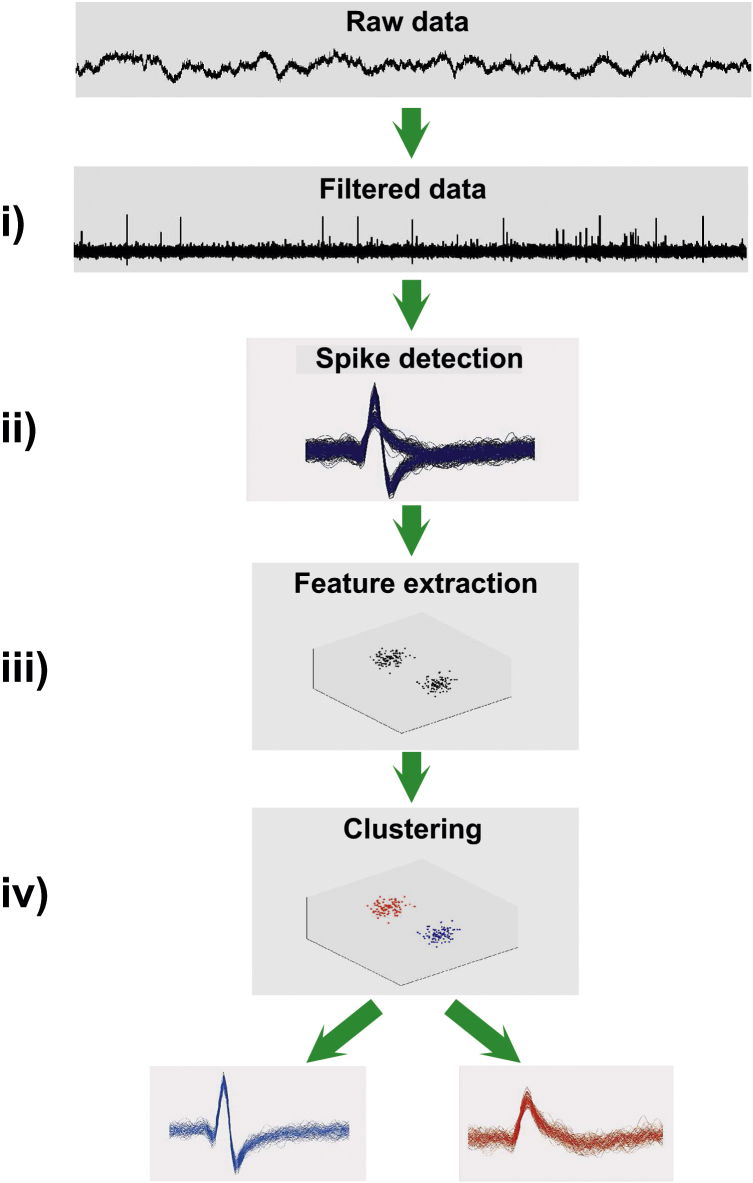
Basic steps for spike sorting. Starting from the recorded raw data, (i) a bandpass filter is applied, e.g., between 300 Hz and 3000 Hz, to keep the most useful part of the spectrum for spike sorting. Next, (ii) spikes are detected, usually using an amplitude threshold applied to the filtered data. In (iii), relevant features of the spike shapes are extracted, achieving a dimensionality reduction. Finally, those features are the input to a clustering algorithm (iv) that performs the classification of the waveforms and associate each cluster to a unit.

*Source*: Adapted from (Quian Quiroga, 2012a).

The classic approach to spike sorting is summarized in Fig. 2. The recorded raw data is first bandpass filtered to facilitate spike detection, which is usually done using an amplitude threshold applied to the filtered data. Afterwards, relevant features of the spike shapes are extracted, which become the input to a clustering algorithm that performs the classification of the waveforms and associate each cluster to a unit. In the following, we discuss the different steps in more detail.

### 2.1 Filtering

As illustrated in Fig. 1, spikes coexist in the raw data with the LFPs and the contribution of each type of signal can be separated by filtering, keeping the high and low frequency activity, respectively. In order to detect the spikes, bandpass filtering allows setting amplitude threshold (see bottom panel in Fig. 1). It should be noticed that the raw data is typically recorded using a hardware acquisition system that includes a first analog causal IIR (infinite impulse response) bandpass filter, e.g., between 0.3 Hz and 7500 Hz. For the purpose of spike detection and sorting, a second digital filter, e.g., between 300 Hz and 3000 Hz, is typically used.

With respect to the type of filter, IIR filters use a feedback path that allows reaching to reach a certain specification (in terms of its frequency response) with a lower order than finite impulse response (FIR) filters, thus being in principle preferred for their efficient hardware and software implementations. However, the feedback path can also be the source of instabilities that should be addressed when the filter is designed and most importantly, causal IIR filters have a nonlinear phase response that can distort the signal of interest. Causal filters are typically used in acquisition systems because each data point is filtered online based on the values of previous data points (a noncausal filter would require knowing future, not yet recorded, values or introducing a delay in the online signal). For the first hardware filter, the nonlinear phase response is not critical because the frequency cutoffs of the filter are far from the frequencies of interest. Regarding the second digital filter, it has been shown that such nonlinear phase responses of the most used causal IIR filters can largely distort the spike shapes (Quian Quiroga, 2009). These filtering artifacts may compromise the distinction between the spikes of pyramidal and inhibitory neurons (Csicsvari et al., 1999, Ison et al., 2011) or interpretations about the relationship between intra- and extra-cellular action potentials (Henze et al., 2000). Moreover, it has been shown that phase nonlinearities not only distort spike shapes but can actually change the appearance of signal artifacts and make them look similar to real spikes (Quian Quiroga, 2009). If offline processing is used, the easiest solution is to use zero-phase filtering, i.e., the phase response is zero for all the frequencies, by processing the input signal in both the forward and reverse direction (Oppenheim et al., 1989). However, some applications require online processing, e.g., closed-loop experiments (where the experimental design is contingent upon the neural activity and/or the behavioral performance), or neural prostheses (where a prosthetic device can be controlled based on the neural activity). In these cases, nearly linear phase IIR filters can be used (Nongpiur et al., 2013, Powell and Chau, 1991).

### 2.2 Detection

After filtering, spikes are easily visualized on top of background noisy activity and can be detected, for example, by using an amplitude threshold. If the value of the threshold is too small, noise fluctuations will lead to false positive events, if it is too large, low-amplitude spikes will be missed. A threshold can be set manually, but since the detection tradeoff is related to the signal to noise ratio of the recording, it seems reasonable to look for an automatic threshold as a multiple of an estimate of the standard deviation of the noise, i.e., $\text{threshold}=k*{\hat{\sigma }}_{n}$, where *k* is a constant typically between 3 and 5.

To set an automatic threshold, it is in principle reasonable to use a value based on the standard deviation of the (filtered) signal. However, this naïve approach can lead to high error rates. For detection purposes, the filtered signal *X* can be approximated as a mixture of the noise component *N* (with zero mean and variance $\sigma_{n}^{2}$) and the spike component *S* (with mean *μ*_*s*_ and variance $\sigma_{s}^{2}$), the latter with probability *p*_*s*_. The resulting distribution would be heavy tailed due to the spike component. However, *p*_*s*_ is very small, so the variance of the mixture can be approximated by

$$\sigma_{x}^{2}\approx \sigma_{n}^{2}+\mu_{s}^{2}p_{s}$$

Clearly, using the variance of the mixture as an estimate of the variance of the noise will lead to a larger threshold, particularly in the cases of high firing rate and large amplitude spikes.

To improve this estimate, an alternative approach was proposed (Quian Quiroga et al., 2004). If we assume that the noise component *N* is normally distributed, it can be shown that

$$\sigma_{n}=\frac{\text{median}(|N|)}{0.6745}\text{,}$$

where the denominator comes from the inverse of the cumulative distribution function for the standard normal distribution evaluated at 0.75. In this case, the fact that *p*_*s*_ is very small leads to the spike component not affecting much the median absolute deviation, i.e., median(|*X*|) ≈ median(|*N*|). Therefore, the estimate used to compute the threshold is

$${\hat{\sigma }}_{n}=\frac{\text{median}(|X|)}{0.6745}$$

This explains why an estimate based on the median absolute deviation of the filtered signal is much more robust than one using the standard deviation estimate. In fact, this improvement has been shown using simulations (Quian Quiroga et al., 2004) and real data (Quian Quiroga, 2007), even if the noise distribution might deviate from Gaussian.

To improve detection performance some sorting algorithms include an extra transformation to the filtered signal before thresholding, e.g., applying an energy operator (Bestel et al., 2012, Rutishauser et al., 2006), the continuous wavelet transform (Nenadic and Burdick, 2005), or using fuzzy and probability theories (Azami et al., 2015). In addition, given that the noise distribution could be nonstationary, it is possible to compute the estimate using blocks of data, e.g., 5 min long. Then, the threshold will be less affected by changes in the noise distribution, avoiding an increase in the detection error rate.

After detection, the next step is to store the putative spike waveforms (∼2 to 3 ms long), which will be the input to the sorting algorithm. In some cases, alignment to the peak of the waveforms can improve the classification process, depending on the features used (see next subsection). But before doing that the waveforms should be interpolated to improve the detection of the peak, e.g., using cubic splines, and afterwards they could be decimated back to the original sampling rate.

### 2.3 Feature extraction

First spike sorting algorithms separated units based on the amplitude of the spikes. Although this can be implemented in a fast and simple way, nearby neurons can have the same peak amplitude but with a different shape (see clusters 2 and 3 in Fig. 1). To improve this, ‘window discriminators’ were introduced, where one or more time-amplitude windows are defined and the waveforms crossing them are assigned to a particular unit (Quian Quiroga, 2007). Even though implementing window discriminators is relatively straightforward and can be done online, this requires manual intervention and it is not practical when a large number of electrodes are used. Furthermore, windows may need to be readjusted during an experiment due to nonstationarity of the recordings and the consequent changes in the spike shapes (see Section 2.5).

Another simple approach relies on choosing template spike shapes for each unit and assigns the detected waveforms via template matching, based on a particular distance metric (Gerstein and Clark, 1964). However, this approach also requires manual intervention and readjustment of the templates when nonstationarities occur. In addition, sparsely firing neurons may be missed, especially if the particular input (or a particular behavior) that elicits the firing of the neuron is not present while the windows/templates are set (Quian Quiroga, 2007).

A different approach involves capturing features from the spike shapes that will be later used for clustering the waveforms. For example, the peak amplitude and width of the spikes can be input to a clustering algorithm (Lewicki, 1998). However, it has been shown that these features are relatively poor for differentiating spike shapes (Quian Quiroga et al., 2004). In general, the more discriminative features we have, the better the ability to distinguish the different spike shapes. If we store *M* samples for each waveform, the spike shapes can be represented as points in an *M*-dimensional space. The complexity of clustering in such a high dimensional space calls for a *dimensionality reduction*. The goal is to keep only the features that help the classification, given that eliminating inputs dominated by noise can improve clustering outcomes (Quian Quiroga, 2007). The main issue is how to choose the minimal set of features that yields the best discrimination.

One of the most common feature extraction and dimensionality reduction methods is principal component analysis (PCA) (Abeles and Goldstein, 1977, Harris et al., 2000, Lewicki, 1998, Shoham et al., 2003). PCA gives an ordered set of orthogonal vectors that capture the directions of largest variations in the data and any waveform is represented as a linear combination of the principal components. Using only the first *K* components can account for most of the energy in the data, so its associated scores can be used as input to a clustering algorithm. This way, the dimensionality reduction is achieved by going from an *M*-dimensional space to a *K*-dimensional space, with *K* ≪ *M* (typically, *K* equals 2 or 3).

The most used alternative to PCA for feature extraction is wavelets (Hulata et al., 2002, Quian Quiroga et al., 2004, Takekawa et al., 2010). The wavelet functions (smooth and quickly vanishing oscillating functions) are formed by dilated (contracted) and shifted versions of a “mother wavelet” (Mallat, 1999). The detected waveforms are then convolved with the wavelets leading to a set of coefficients that represent the signal decomposition. Therefore, a large correlation between the signal and the wavelet at a certain time and scale shows how well the wavelet can be used as a local approximation of the signal. This way, very localized shape differences in the spikes of individual units can be discerned with a few wavelet coefficients. The information about the shape of the spikes will be distributed in several wavelet coefficients (unlike PCA, where most of the energy is concentrated in the first three components) and it has been shown that better performance can be achieved with respect to PCA (Quian Quiroga et al., 2004). This difference is mainly due to the fact that: (i) unlike PCA, wavelet coefficients are localized in time and (ii) directions of maximum variance chosen with PCA are not necessarily the ones of maximum separation between spike shapes.

The key issue with wavelets is how to automatically select coefficients that best distinguish the different spike shapes. For this, the following criterion has been proposed (Quian Quiroga et al., 2004): if the data contains more than one unit, a wavelet coefficient that can separate the different spikes shapes should have a multimodal distribution. This can be quantified using the Lilliefors modification of a Kolmogorov–Smirnov (KS) test for normality to achieve the dimensionality reduction by selecting the first 10 coefficients with the largest deviation from normality (Quian Quiroga et al., 2004).

The criterion of a multimodal distribution applies not only to wavelets coefficients but to any feature. With this in mind, it has been proposed (Bestel et al., 2012) to extract a variety of geometric, wavelet and PCA-based features, introducing a unidimensional score to automatically select a feature subset to sort spikes.

### 2.4 Clustering

The final step of the spike sorting process is to group the points in the feature space into clusters, with each cluster associated to a different neuron. With manual cluster-cutting, the user defines the boundaries for the different clusters (Gray et al., 1995). However, this relies on analyzing different two- or three-dimensional projections of the feature space, which can be very time consuming. Moreover, manual clustering introduces errors due to both the limited dimensionality of the cluster cutting space and human biases (Harris et al., 2000, Pedreira et al., 2012).

Alternatively, a few algorithms were constructed within the framework of Bayesian clustering and classification (Lewicki, 1998). For this, clusters are assumed, in general, to have a Gaussian distribution, based on the claim that for a given cluster the spike variability is determined only by additive and Gaussian stationary background noise. If *C* clusters are assumed, the parameters of the resulting mixture of Gaussians can be estimated using an Expectation Maximization procedure (Harris et al., 2000, Pouzat et al., 2002). As *C* is actually unknown, several models (with different values of *C*) can be estimated and a penalty function is introduced to penalize overfitting, by discouraging models with a large number of parameters (Schwarz, 1978). A related approach involves using infinite Gaussian mixture models, so that the effective number of clusters/units is decided in an unsupervised way (Wood and Black, 2008).

One problem with the ‘mixture of Gaussians’ approach is that there are several experimental conditions that lead to clusters with non-Gaussian shapes (see below). In those cases, the structure of the data can be very difficult to capture with a mixture model, making it challenging to predict the number of units and to make accurate classifications (Lewicki, 1998). In fact, methods assuming Gaussian distributions tend to overcluster by fitting several multivariate Gaussians to a single non-Gaussian cluster. One alternative is to use a mixture of distributions with a wider tail, e.g., *t*-distributions (Shoham et al., 2003). Another alternative is to use hierarchical clustering, where the data is first grouped into a large number of clusters, which are then merged according to similarities in the spike shapes and the interspike interval distribution (Fee et al., 1996).

Yet another option is to use nonparametric clustering algorithms, e.g., based on nearest neighbor interactions. One example is super-paramagnetic clustering (SPC), which has been successfully applied for spike sorting (Quian Quiroga et al., 2004). This stochastic algorithm uses a single parameter (the ‘temperature’) to group the spikes into clusters. Following the analogy with statistical mechanics, for low temperatures all the data is grouped into a single cluster and for high temperatures the data is split into many clusters with few members each. However, there is a middle range of temperatures (the super-paramagnetic regime) where clusters of a relatively large size, corresponding to the different single units, are captured. Being a nonparametric algorithm, SPC does not assume that clusters are nonoverlapping, that they have low variance, or that they follow a Gaussian distribution. Since SPC can leave points unclassified, a final template matching step is generally used to complete the classification result. This approach has already being shown to cope well with sparsely firing neurons and the presence of clusters with largely different sizes (Rey et al., 2014). Moreover, to better optimize the identification of clusters with different sizes, a sequential application of SPC has been proposed (Ott et al., 2005).

Alternative classification algorithms have been tested in the context of spike sorting, such as neural networks approaches and support vector machines. An overview of published spike sorting algorithms can be found in (Bestel et al., 2012).

### 2.5 Other issues

#### 2.5.1 Validation of results

Once a sorting solution is obtained, it might be useful to quantify the degree of confidence on the results, based on the cluster separation. When spikes from two different neurons are incorrectly classified as a single cluster, it is possible to have spikes with interspike intervals below the minimum refractory period of the neuron (about 2–3 ms). If the proportion of refractory period violations is significant, it can be seen as a measure of poor isolation of the single units (however, the opposite is not true, i.e., the absence of refractory period violations does not guarantee single neuron clusters). Alternatively, it has been proposed that a non-uniform variance of the spike shape is also a measure of poor unit isolation (Pouzat et al., 2002). The separations between clusters of spikes can also be quantified (Hill et al., 2011, Schmitzer-Torbert et al., 2005, Swindale and Spacek, 2014) and used to decide whether or not two clusters should be merged—although if the quality metrics are based on certain model hypotheses (e.g., Gaussian distributions) they might be of relative use when these hypotheses are not met. Still, in most cases the final sorting outcome is given by the user, which does not only have an impact on the overall error rate but can also exhibit subjective biases, depending on the relative importance given to false positive and false negative errors (Harris et al., 2000).

#### 2.5.2 Tetrodes

Tetrodes are 4 close-by recording electrodes that are used to observe the same set of neurons from different sites. They can be constructed by twisting together four small individually insulated microwires or by placing four contacts in a neural probe with a typical separation of 25–50 μm. The use of tetrodes, or more generally of polytrodes, has been shown to increase dramatically the single cell yield (Gray et al., 1995, Harris et al., 2000). Also, they provide better sorting quality, since an ambiguous separation from one channel can be disentangled using the information from another nearby channel (Quian Quiroga, 2012a).

To perform spike sorting from tetrode recordings, the peak amplitude or the first principal components for each of the channels are used as inputs to the clustering algorithm. Alternatively, clustering of tetrode data can be done by concatenating the spike shapes of the four channels (Quian Quiroga, 2007). With any of these methods, a single tetrode can isolate up to a couple dozens of neurons, something that is important, for example, to study properties of cell assemblies (Buzsáki, 2004).

#### 2.5.3 Overlapping spikes

When two nearby neurons fire synchronously or with a small delay, their spikes will overlap in time. If the delay is long enough, the appearance of double peaks might be enough to recognize the two spikes in the waveform. However, if the spikes are too close they can create complex waveforms, which in turn will difficult the cluster isolation and may even give rise to new clusters. The problem of overlapping spikes is one of the most challenging issues in spike sorting and although several methods have been proposed (Hulata et al., 2002, Lewicki, 1994, Pillow et al., 2013, Prentice et al., 2011, Takahashi et al., 2003), there is no optimal approach to deal with it. In this regard, the use of tetrodes or multielectrode probes can be helpful to deal with this issue since what appears as an overlap on one channel might be an isolated unit on another (Lewicki, 1998).

#### 2.5.4 Bursting neurons

Some neurons generate fast sequences of action potentials called bursts. Typically, the amplitude of the spikes across the sequence decreases and they may be assigned to different clusters. When all these spikes are actually merged, peaks in the interspike interval distribution can be observed, associated to the small time between spikes within a burst. A particular pattern with peaks at about 3 ms will also be observed in the cross-correlogram if the clusters are analyzed separately, which gives a clear indication that these clusters correspond to the same bursting neuron. In general, the key information to merge clusters into single burst is the reliable timing between the sequences of spikes.

#### 2.5.5 Electrode drifts

If the neural tissue moves, the electrode can (slowly) drift to a different position. A typical case is the retraction of the cortical tissue after implanting an acute electrode (for this reason, researchers usually wait 30 min to 1 h after implantation of the electrodes for the recording to stabilize). During a recording session this might translate as a (gradual) change in the shape of the spikes associated to a single unit. Clearly, the associated cluster for the neuron will have a non-Gaussian distribution. In fact, clusters might split, or even appear or disappear altogether. One possibility to deal with this issue is to plot a certain feature (e.g., the peak amplitude) as a function of time in the recording. In addition, when dealing with recordings that might last for several days or weeks, significant changes might take place in the surroundings of the electrode, e.g., due to growth of glia cells. In this respect, some sorting algorithms have considered modeling electrode drifts explicitly to improve their performance (Bar-Hillel et al., 2006), whereas other algorithms not assuming a particular shape of the clusters can cope with these nonstationarities as long as the changes are not too fast (Quian Quiroga et al., 2004).

### 2.6 Alternative approaches

Departing from the basic steps described in Fig. 2, alternative spike sorting algorithms avoid the detection and/or feature extraction and clustering stages. For example, template matching is a particularly appealing when online sorting is necessary, given its simplicity and low computational cost. Once the templates are computed with an offline sorter, they can be used for template matching, in order to classify each new detected spike online (Franke et al., 2012). In line, Rutishauser et al. (2006) proposed to compute templates as the spikes are detected, thus avoiding the use an offline sorter. Template matching has also been used to perform spike sorting on recordings from retinal multi-electrode-arrays (MEAs) (e.g., Marre et al., 2012). However, it should be noticed that due to the particulars of retinal preparations, some strategies that are suitable for retinal recordings might not translate well into other scenarios, e.g., high count electrode in vivo recordings from cortical areas. Within spike sorting algorithms that do not use the standard structure of filtering, detection, feature extraction and clustering, it is worth mentioning a model-based approach to sort based on estimations of the most probable time patterns (Ekanadham et al., 2014), or the use of the OPTICS algorithm (Ankerst et al., 1999, Schneider and Vlachos, 2013) directly applied to the detected waveforms (Prentice et al., 2011) to create an ordered sequence of spikes.

## 3 Modeling extracellular recordings to validate spike sorting algorithms

We previously discussed different metrics used to validate the outcomes of spike sorting algorithms. However, whether two clusters should be merged or whether an obtained cluster does not correspond to a well isolated single unit is a decision that is ultimately left to the user, and is therefore subjective in nature.

In order to test the performance of spike sorting algorithms objectively, there is a need for ground truth, i.e., knowing the identity of the neurons generating each detected spike. One possibility is to look at simultaneous intracellular and extracellular recordings (Harris et al., 2000, Wehr et al., 1999), but the number of neurons that can be simultaneously recorded is not large enough (e.g., up to 4 in Anastassiou et al., 2013) with respect to the number of neurons than can be simultaneously recorded with an extracellular electrode or tetrode. This limitation calls for a different approach.

The most viable alternative is to use synthetic data obtained with computational models. Simulated datasets obtained this way can be used to objectively quantify and compare the performance of different sorting algorithms (Einevoll et al., 2012). The modeling challenge is then to reproduce the most relevant features of extracellular recordings. A simple strategy is to simulate recordings by adding spikes to Gaussian noise (Lewicki, 1994). This approach is very fast and easy to implement, but it fails to reproduce important features of the real recordings, such as the presence of non-Gaussian clusters, multiunit activity and spectral similarity between noise and spikes, which might be critical for spike sorting. In electrophysiological recordings, potential noise sources include true (Johnson) noise in the electrode and electronics, electrical pickup from the environment, as well as background activity from distant neurons (Fee et al., 1996).

The approach in (Martinez et al., 2009) uses a database of previously recorded spike shapes placed at random times. Single unit activity was simulated to have an amplitude between 1.5 and 4 times the level of the detection threshold, as in real recordings. Multiunit activity gave rise to non-Gaussian clusters (because they reflect the activity of more than one neuron and the amplitude distribution is truncated by the detection threshold) and was generated by mixing the activity of ∼600 spike shapes using amplitudes uniformly distributed between 0.5 and 1.5 times the level of the detection threshold. In addition, the background noise was generated by superimposing millions spikes shapes with their amplitude inversely proportional to their distance from the electrode tip, and also adding Gaussian noise to simulate the noise introduced by the recording equipment. This way, the characteristics of the noise, such as its amplitude and frequency distribution, arise naturally from the realistic biophysical process of its generation and it is not imposed beforehand. A more detailed approach was used in (Lindén et al., 2014), where morphologically reconstructed neurons were first simulated with compartmental modeling to provide transmembrane currents, e.g., using the program NEURON (Hines and Carnevale, 1997), and were then used to calculate the extracellular potentials. Although this approach can lead to a more realistic model of the extracellular recording, it can be quite computationally intensive.

**Fig. 3:**
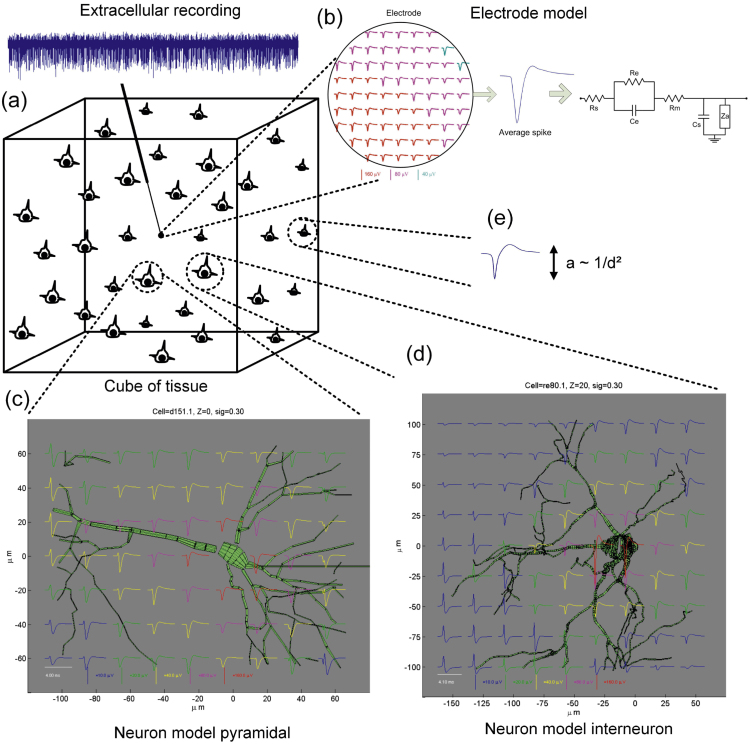
Modeling extracellular recordings. (a) Several neurons are randomly placed in a cube of tissue with the recording electrode. The extracellular recording was obtained by averaging the electric potential over the electrode surface and filtering with the electrode model (b), where Rs is the spreading resistance, Re is the leakage resistance, Ce is the capacitance of the interface, Rm is the metallic resistance, Cs is the shunt capacitance, and Za is the input impedance of the amplifier. The contribution to the electric potential from neurons nearby the electrode (less than 150 μm) was computed with a detailed compartmental model using the software NEURON (Hines and Carnevale, 1997). (c) and (d) show examples from a pyramidal neuron and interneuron, respectively. In addition, the background noise was generated by adding contributions from further away neurons (e), using previously recorded spike shapes from a database with an amplitude scaled by the squared distance to the electrode.

*Source*: Adapted from (Camuñas-Mesa and Quian Quiroga, 2013).

One problem of the approach from (Martinez et al., 2009) is that the position and shape of the electrode affects not only the amplitude but also the shape of the spikes from nearby neurons. At the same time, using the approach from (Lindén et al., 2014) to simulate 1 mm^3^ of brain tissue might involve running a detailed model for ∼300,000 neurons, which has a very large computational cost. To cope with these issues, a hybrid approach was introduced in (Camuñas-Mesa and Quian Quiroga, 2013). As illustrated in Fig. 3, the key point is to use a detailed compartmental model to simulate the contribution of neurons near the recording electrode (Gold et al., 2007), and previously recorded spike shapes to generate the background noise, as in (Martinez et al., 2009). By assuming a resistive medium, the amplitude of spikes from far-away neurons was normalized by the squared distance to the electrode (dipole approximation), without affecting their shape. For the detailed model, a line source approximation was used, locating the transmembrane net current for each neurite on a line along its center. The distance separating the two zones (for detailed modeling and background noise) was set to 150 μm, given that for larger distances the variability of the spike shape was negligible and the amplitude decayed as the inverse square of the distance, tightly following the dipole approximation. Interestingly, this is approximately the distance that separate the detected spikes from background noise, as has been shown with simultaneous intra- and extra-cellular recordings at different distances from the soma (Henze et al., 2000). Furthermore, given that the electrode tip is not a single point source, the effects of finite-size electrodes were also modeled by averaging across their surface and considering capacitance filtering effects. The importance of not considering the electrode as point sources is stressed by the fact that the shape and size of the electrodes affect the recordings. In particular, the larger the tip of the electrode the greater the number of neurons recorded. If the electrode tip is too large, it will be impossible to isolate any one particular neuron. If it is too small, it might be difficult to detect any signal at all (Camuñas-Mesa and Quian Quiroga, 2013). This hybrid model offers a platform to create synthetic datasets for testing spike sorting algorithms, together with new electrode designs, as it replicates the most relevant features of real extracellular recordings, without an excessive computational cost. Moreover, it also provides a good platform to objectively and systematically test algorithms to deal with large electrode arrays, with different size and separation between them (see also Section 6).

## 4 On-chip solutions

The neural activity measured by the implanted electrodes is typically transmitted through wires to a recording system. However, the need of wires passing through the scalp increases the chance of infections. Moreover, the tethered connection to the external amplifier and/or acquisition system imposes mobility restrictions to the subject, limiting the experimental setups and clinical studies that can be carried out. Using a wireless interface would be ideal to overcome these issues. In addition, it is a very appealing solution for the neuroscience community in order to perform animal experiments in more ecological conditions. Furthermore, the design of low-power implantable wireless interfaces will also boost the development of brain-machine interfaces (BMIs) to control prosthetic devices with brain activity, which could be used for therapeutic purposes (Homer et al., 2013, Nicolelis and Lebedev, 2009).

What type of information do we need to transmit through the wireless link? A first approach is to transmit the broadband signal (containing both the spike and LFP activity). However, this solution is in principle prone to bandwidth limitations (restricting the number of channels that can be transmitted) as well as power requirements. Still, recent advances in this area show promising results. Yin et al. (2014) introduced a new wireless platform with the capacity to transmit the full broadband signal, whose performance was similar to the one obtained with wired recordings. The device was successfully tested to study locomotion dynamics of a monkey moving on a treadmill and sleep-wake transitions of a monkey while they were in their home cages (representing good examples of the potential applications of wireless technology in more ecological conditions). However, it was assembled outside the head and its battery needs replacement after less than 2 days of continuous monitoring of 100 channels.

An alternative approach is to perform most of the computations close to the recording electrodes, reducing considerably the amount of data that needs to be transmitted through the wireless link. In fact, spike sorting could be done on-chip and only the timestamps (and the label of its associated unit) would have to be transmitted wirelessly. However, such an algorithm will have to be completely unsupervised, online, with low power consumption and computational cost (to be implemented on a chip).

Yet another solution is to follow a hybrid approach. Spike shapes can be first transmitted wirelessly to a receiver so that spike sorting (with all its heavy calculations) is performed offline in a computer. Then, templates can be defined for each channel and send back to the chip, so that new spikes can be sorted automatically ‘on-chip’ using template matching (Navajas et al., 2014, Schwarz et al., 2014). In the latter work, volumetric recording probes with up to 512 channels were used in monkeys. However, the device consumed approximately 2 mW per channel, with a range of up to 3 meters.

In order to improve this solution even further, a systematic study used simulated recordings to assess the minimum requirements for an on-chip spike sorting implementation, that gives accurate results and is efficient in terms of computational cost and power consumption (Navajas et al., 2014). During a ‘Template Building’ stage, a low-power implantable platform filtered the data, performed analog to digital conversion, and detected the spikes in real time. The authors showed that by reducing the sampling rate from 28 kHz to 7 kHz and the resolution from 16 bits to 10 bits (with a resulting signal resolution of 0.97 uV/bit) it was still possible to achieve a good spike sorting performance. With respect to the template matching (on-chip) stage, the authors tried several metrics and showed that the squared Euclidean distance exhibited the best relationship of performance versus complexity for hardware implementation, requiring only a window size of 0.5 ms around the peak for the comparison. In addition, a good peak alignment was shown to be important before template matching, which was done using a low power alternative to the classic approach of upsampling before finding the peak (Paraskevopoulou and Constandinou, 2011). Overall, this approach was validated using real extracellular recordings and a drop in performance of only ∼20% was achieved by reducing the amount of data needed to be processed by ∼85%. Moreover, it was estimated that such an approach would only need 2.24 mW for 100 channels (with 100 neurons firing at 1 Hz) on a 10 Mbit/s maximum channel capacity. Another important advantage is that the whole device can be implanted subcutaneously (with the battery being recharged wirelessly), which has an impact on the well being of the animal as they do not have direct access to the device.

### 4.1 Is spike sorting necessary?

All the issues arising to implement on-chip spike sorting solutions naturally lead to the question of whether it is really necessary to use large resources in sorting the spikes, or whether a simple unclassified set of detected spikes suffices. In the context of BMI applications, it has been argued that it might not be necessary to use sorted spikes into a decoder (Fraser et al., 2009, Ganguly et al., 2011, Kloosterman et al., 2014). However, Todorova et al. (2014) showed that for the purpose of decoding, crude and simple automated spike sorting (e.g., assigning waveforms to clusters with boundaries given by the quartiles of waveform amplitudes observed during a training period) was almost as effective as expert sorting (and in some cases even outperformed the traditional model-based Gaussian clustering) and better than decoding without spike sorting. In fact, improvements with spike sorting have also been reported in the context of BMIs. Ganguly et al. (2011) showed that by monitoring individual neurons, two populations (direct and indirect) could be identified, exhibiting different changes in the modulation of the neural activity during the process of learning neuroprosthetic control.

In other scenarios, the quality of the spike sorting might be essential. It has been shown that the identity of a visual stimulus can be decoded using the firing rates of neurons recorded in the human medial temporal lobe and that spike sorting improves decoding performance by 10% on average, going up to 50% in some sessions (Quian Quiroga et al., 2007). This improvement is due to the fact that in general these neurons have a very sparse firing and present a very high stimulus selectivity, which is typically different for close-by neurons recorded from the same electrode (Rey et al., 2014).

## 5 Limitations of current spike sorting algorithms

In the last few years it has been stressed that there is a discrepancy between the number of neurons identified from extracellular recordings and the one supposed to be found based on biophysical and anatomical considerations. By recording simultaneous intra- and extra-cellular recordings from the rat hippocampus, Henze et al. (2000) showed that single neurons can be observed up to a distance of 50 μm from the cell body. Then, there should be about hundred neurons within a sphere with this radius that we should be identifying with single channel recordings. However, the number of neurons reported with single recording channels is generally below ten.

A number of reasons have been suggested to explain the discrepancy between the number of neurons we should be observing and the ones that are actually observed (Buzsáki, 2004, Shoham et al., 2006). Some of them include tissue damage caused by the insertion of the electrodes in the recording area (Claverol-Tinture and Nadasdy, 2004) or the electrical insulation caused by the substrate of the probe (Moffitt and McIntyre, 2005). Alternatively, it has been argued that this is due to the low firing rate of relatively large number of neurons (referred as silent neurons). The presence of nearly silent neurons (with very low baseline rates) has been reported in recordings from the medial temporal lobe in humans (Rey et al., 2014) and, more generally, in the mammalian neocortex and hippocampus, as they are silent during awake states but can be observed under anesthesia (Shoham et al., 2006). Moreover, in the human recordings it has been shown that such a low baseline activity comes together with high stimulus selectivity (Ison et al., 2011) and these neurons are likely to be missed unless the right stimulus is shown during the experiment. Interestingly, some of these nearly silent neurons have been related to high level cognitive processes; for example, playing a determinant role in the formation of memories (Quian Quiroga, 2012b, Rey et al., 2014).

**Fig. 4:**
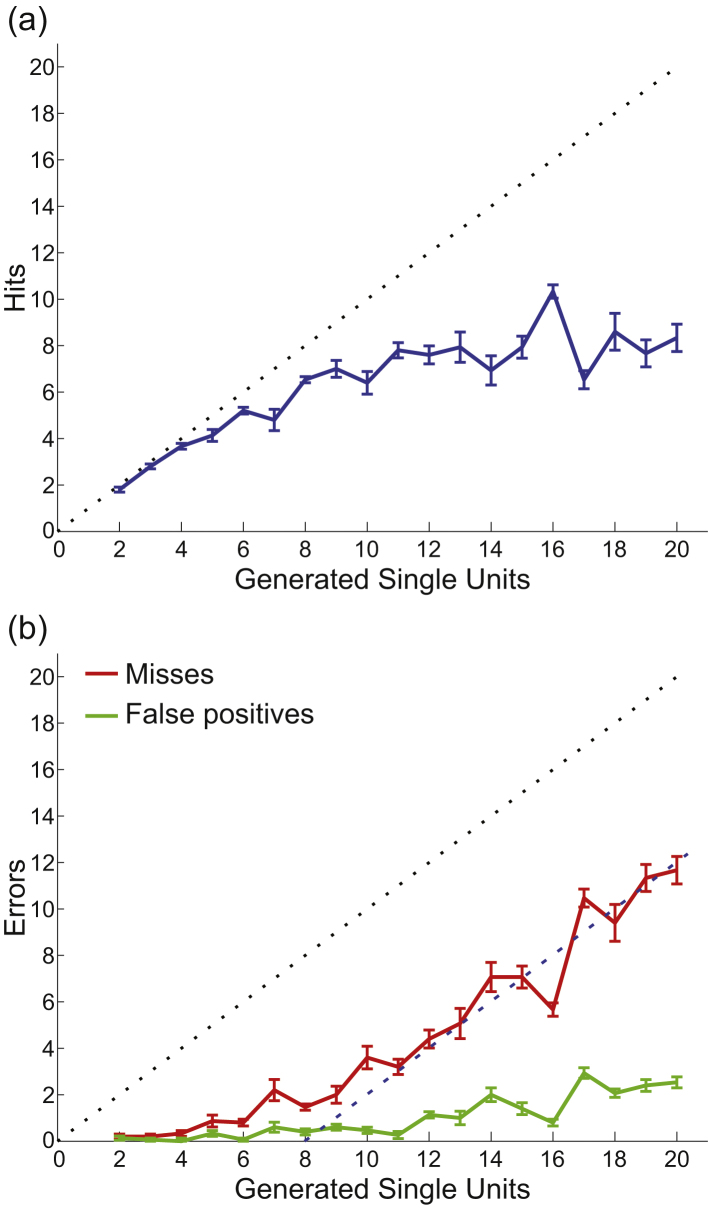
Spike sorting performance with increasing number of neurons. Average results for 3 users that sorted 95 different simulations containing between 2 and 20 different units (the dataset can be downloaded from http://bioweb.me/CPGJNM2012-dataset). (a) Average number of hits. The performance remains close to the ideal (the diagonal line) for up to 5 or 6 units in the simulation. When the units in the simulations keep increasing, the number of units correctly identified by the user deviated from the ideal and pointed toward an asymptotic value between 8 and 10 units. (b) Errors in the sorting process detailed as false positives (green; units sorted that do not correspond to a generated unit) and misses (red; units generated that have no corresponding cluster). While the false positives remained low for all cases, the misses followed the asymptotic line corresponding to 8 correct units detected (dashed line at *Y* = *X* − 8). Error bars denote SEM. (For interpretation of the references to color in this figure legend, the reader is referred to the web version of this article.)

*Source*: Adapted from (Pedreira et al., 2012).

Besides the issue of silent neurons, another factor adding to the relatively low number of recorded neurons has to do with limitations of current spike sorting algorithms. In fact, Pedreira et al. (2012) evaluated the ability of spike sorting algorithms to identify a varying number of neurons (from 2 to 20) in simulated recordings. Fig. 4 shows a summary of the results, where the sorting was performed independently and blindly by three expert operators using Wave_clus (Quian Quiroga et al., 2004). For the number of neurons typically reported in the literature (up to 4) the performance of the algorithm was, as expected, almost perfect. Most of the units were correctly identified (hits) and no spurious clusters (false positives) were detected. However, when the number of units was higher, the sorting performance decreased, reaching a maximum of about 8 units. Interestingly, the units that were more frequently missed were the ones with a lower firing rate, thus being the most silent ones, in agreement with previous claims (Shoham et al., 2006).

## 6 Conclusions and future challenges

One of the most striking facts about spike sorting methods is that, despite having been used for decades, there is no clear agreement in the field about spike sorting standards. To compare different methods, we have argued in Section 3 that simulated signals present the ideal test-bench. The approaches to perform these simulations are varied, but in most cases they comprise the activity of a few units superimposed to some background noise. A recent work by Camuñas-Mesa and Quian Quiroga (2013) went a step beyond and provided a more realistic scenario, including a three-dimensional configuration of the recording space. This allows setting the basic characteristics of the neural tissue (such as the density of neurons) as well as the location and size of the recording electrodes. The 3D nature of the model also provides a perfect frame for the study of algorithms to deal with multielectrode probes, since the correlation of amplitudes across contacts would match the one expected in the probe in neural tissue.

When ground truth is available, the performance of an algorithm is usually evaluated in terms of the number of hits and misses it produces. In particular, we have shown that current algorithms isolate a maximum of 8–10 units per channel, when up to 20 neurons were present in the recording (Pedreira et al., 2012). This surprisingly poor performance compared to previous reports can be attributed to the fact that previous simulations considered only relatively simple scenarios, with only a few (e.g., up to 3) neurons per recording. We propose that new spike sorting algorithms have to deal with recordings of a much higher complexity and we therefore share the simulated dataset used in (Pedreira et al., 2012), to test alternative approaches. The data can be downloaded from http://bioweb.me/CPGJNM2012-dataset.

Going beyond the quantification of sorting accuracy in terms of misses and false positives, some works have focused on how the accuracy of the sorting process affects other measures, such as spike synchrony (Pazienti and Grün, 2006), rate code estimates (Ventura, 2009), and neuronal correlations (Cohen and Kohn, 2011, Ventura and Gerkin, 2012). Further work on this line can in turn highlight what type of sorting errors are the most detrimental when computing different measures (e.g. the bias introduced in computing firing rates might be different to the one associated to synchrony/coincident spiking).

The development of spike sorting algorithms should go hand in hand with developments in recording techniques. Tetrodes have been used for over 25 years and other type of polytrode configurations and silicon probes have been developed in the last 10 years (Blanche et al., 2005, Buzsáki, 2004, Csicsvari et al., 2003), allowing simultaneous recording of neuronal activity in the various cortical layers. In addition, large MEAs with up to thousands electrode sites are already starting to be used for recording in retinal patches (Litke et al., 2004), cell cultures (Lambacher et al., 2011), or brain slices (Frey et al., 2009). Furthermore, large number of channels are currently used to record from local circuits in behaving animals (Berenyi et al., 2014). The improvements in the probes’ fabrication and the degree of integration in electronics available nowadays have made possible a more advanced approach to electrophysiological recordings, compared to the traditional insertion of tungsten electrodes. In addition to the obvious advantages of recording from far many more units within the same structure, new recording techniques open the door to the ability of recording simultaneously from entire processing units, such as a cortical column. In fact, we seem to be at the verge of a qualitative change in terms of the number of sites from which we can simultaneously record, going up to thousands (Alivisatos et al., 2013, Khodagholy et al., 2013, Khodagholy et al., 2014).

The use of large multichannel electrodes imposes, however, a number of new challenges that need to be urgently addressed. The amount of data generated from such recordings is very large, and traditional feature extraction techniques for dimensionality reduction still lead to very large dimensional spaces where sorting would be done (Einevoll et al., 2012). In fact, the use of hundreds or thousands of simultaneously recorded electrodes with a potentially small separation between them (less than 50 μm) introduces large amounts of redundancy and complementary information. The key issue in this respect is how to select, separate and combine information from the different channels, an issue that is only starting to be addressed (Ekanadham et al., 2014, Kadir et al., 2014, Pillow et al., 2013, Swindale and Spacek, 2014).

Another topic of large interest to the neuroscience community is how to develop spike sorting algorithms on a low power chip, to enable wireless transmission of the data and more ecological and secure conditions to perform experiments with animals. In this respect, we have described, on the one hand, the minimum signal requirements to achieve a reasonable sorting performance (reducing about 85% the data processing needs) and, on the other hand, a hybrid strategy to perform an online spike sorting on-chip via template matching. On-chip data processing together with wireless transmission of the results is important for long term recordings in the context of experiments to study sleep (e.g., memory consolidation), to continuously monitor neural activity for clinical purposes (e.g., epilepsy), or BMIs that are starting to be used in clinical trials with human patients (Homer et al., 2013). In this sense, it is also important to provide spike sorting algorithms with tools that would help tracking neurons over long periods of time (Vaidya et al., 2014).

Summarizing, progress in neuroscience depends on the simultaneous recording of large neural populations. This is within reach with technological advances in the design of new electrode probes and acquisition systems, and cries out for the development of relatively fast, multichannel and fully automatic spike sorting techniques to cope with the recorded data.

## Conflict of interest

The authors declare that there are no conflicts of interest.
